# Neuronal Ig/Caspr Recognition Promotes the Formation of Axoaxonic Synapses in Mouse Spinal Cord

**DOI:** 10.1016/j.neuron.2013.10.060

**Published:** 2014-01-08

**Authors:** Soha Ashrafi, J. Nicholas Betley, John D. Comer, Susan Brenner-Morton, Vered Bar, Yasushi Shimoda, Kazutada Watanabe, Elior Peles, Thomas M. Jessell, Julia A. Kaltschmidt

**Affiliations:** 1 Neuroscience Program, Weill Cornell Graduate School of Medical Sciences, New York, NY 10065, USA; 2 Developmental Biology Program, Sloan-Kettering Institute, New York, NY 10065, USA; 3 Howard Hughes Medical Institute, Kavli Institute of Brain Science, Departments of Neuroscience, Biochemistry and Molecular Biophysics, Columbia University, New York, NY 10032, USA; 4 Weill Cornell/Rockefeller/Sloan-Kettering Tri-Institutional MD-PhD Program, New York, NY 10065, USA; 5 Department of Molecular Cell Biology, The Weizmann Institute of Science, Rehovot 76100, Israel; 6 Department of Bioengineering, Nagaoka University of Technology, 1603-1, Kamitomiokamachi, Nagaoka, Niigata 940-2188, Japan; 7 Nagaoka National College of Technology, 888, Nishikatakaimachi, Nagaoka, Niigata 940-8532, Japan

## Abstract

Inhibitory microcircuits are wired with a precision that underlies their complex regulatory roles in neural information processing. In the spinal cord, one specialized class of GABAergic interneurons (GABApre) mediates presynaptic inhibitory control of sensory-motor synapses. The synaptic targeting of these GABAergic neurons exhibits an absolute dependence on proprioceptive sensory terminals, yet the molecular underpinnings of this specialized axoaxonic organization remain unclear. Here, we show that sensory expression of an NB2 (Contactin5)/Caspr4 coreceptor complex, together with spinal interneuron expression of NrCAM/CHL1, directs the high-density accumulation of GABAergic boutons on sensory terminals. Moreover, genetic elimination of NB2 results in a disproportionate stripping of inhibitory boutons from high-density GABApre-sensory synapses, suggesting that the preterminal axons of GABApre neurons compete for access to individual sensory terminals. Our findings define a recognition complex that contributes to the assembly and organization of a specialized GABAergic microcircuit.

## Highlights

• Sensory Ig/Caspr4 complex directs inhibitory synapse formation in mouse spinal cord
• Eliminating NB2 results in a reduced number of GABApre-sensory synapses
• Quantitative modeling suggests competition for formation of axoaxonic synapses
• Role for a contactin/Caspr complex in central synapse formation

## Teaser

The mechanisms that confer synaptic specificity in mammalian circuits remain unclear. Ashrafi et al. describe a selective role for contactin-Caspr recognition in directing the formation of a presynaptic inhibitory microcircuit in the developing mouse spinal cord.

## Introduction

In many regions of the mammalian CNS, inhibitory microcircuits are wired with high precision, fine-tuning synaptic input and modulating neural output (Stepanyants et al., 2004). The assembly of functional inhibitory microcircuits can be considered in several independent steps: the selection of membrane subdomains on specific neuronal targets, the assignment of appropriate synaptic innervation densities, and the regulation of transmitter phenotype and level (Williams et al., 2010). How these diverse cellular processes are orchestrated at individual synapses within defined CNS microcircuits remains unclear.

One informative instance of the subcellular targeting of inhibitory synapses is found in primary sensory systems, where sensory terminals serve both as presynaptic structures that innervate recipient CNS neurons and as the postsynaptic target of local inhibitory interneurons at axoaxonic synapses (Rudomin, 2009). Such axoaxonic arrangements provide an anatomical substrate for selective filtering of sensory information (Rudomin and Schmidt, 1999). In the ventral spinal cord, the central terminals of proprioceptive sensory neurons are studded with numerous synaptic boutons that derive from a discrete set of GABAergic inhibitory interneurons, termed GABApre neurons (Betley et al., 2009, Hughes et al., 2005). This set of spinal inhibitory interneurons can be distinguished by expression of the GABA synthetic enzyme glutamic acid decarboxylase-2 (GAD2/GAD65) (Betley et al., 2009, Hughes et al., 2005), an essential determinant of sustained GABA release (Tian et al., 1999). High-level expression of GAD65 in GABApre neurons is directed by a sensory source of brain-derived neurotrophic factor (BDNF) (Betley et al., 2009). Moreover, sensory terminals in the ventral spinal cord represent the sole target of GABApre neurons (Betley et al., 2009), implying stringent recognition specificity in the assembly and organization of this specialized inhibitory microcircuit.

The molecular mediators of stringent axoaxonic specificity have remained unclear, however. In this study, we used mouse molecular genetic approaches to show that the sensory expression of the immunoglobulin (Ig) superfamily protein NB2 (Contactin5) and the contactin-associated protein Caspr4 are required to establish high-density studding of GABApre boutons on proprioceptive sensory terminals. In a complementary manner, two members of the L1 Ig family, CHL1 and NrCAM, are expressed by GABApre neurons and their function is required for the formation of high-density GABApre synapses with sensory terminals. Our findings pinpoint a molecular recognition system that helps to direct the formation of presynaptic inhibitory synapses.

## Results

### NB2 Expression by Proprioceptive Sensory Neurons

To define potential GABApre recognition molecules expressed by sensory neurons we screened 45 transcripts encoding Ig domain-containing proteins for expression in dorsal root ganglia (DRG) and spinal cord at postnatal days (p)5 to p6—the period at which GABApre axons form contacts with proprioceptive sensory terminals (Table S1 available online) (Betley et al., 2009). To explore the idea that incoming GABApre axons recognize receptors on sensory but not motor neurons, we focused our attention on transcripts expressed selectively by proprioceptive sensory neurons.

This expression screen identified four transcripts, *Contactin* (*Cntn*) *3*, *Cntn5*, *Kirrel*, and *Kirrel-3*, each of which was expressed by DRG neurons but not motor neurons. Only two of these, *Kirrel-3* and *Cntn5* were expressed by proprioceptors, as revealed by coexpression of Parvalbumin (Pv) (Table S1) (Arber et al., 2000). Analysis of *Kirrel-3* mutant mice (Prince et al., 2013) did not reveal a GABApre targeting phenotype (unpublished observations), leading us to focus on the potential role of the contactin family (Shimoda and Watanabe, 2009).

**Figure 1:**
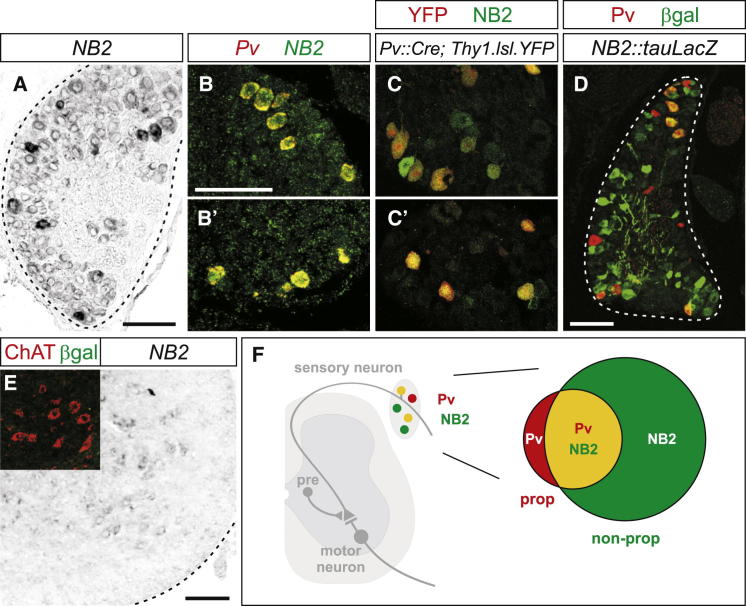
NB2 Expression in Sensory Neurons (A) *NB2* transcript expression in p6 DRG neurons. Scale bar represents 100 μm. (B and B′) *Pv*^*ON*^ (red) proprioceptive sensory neurons express *NB2* (green) in p6 DRG neurons. Scale bar represents 100 μm. (C and C′) YFP^ON^ (red) proprioceptive sensory neurons in p6 *Pv::Cre; Thy1.lsl.YFP* mice express NB2 (green). In *Pv::Cre; Thy1.lsl.YFP* mice, YFP is expressed in Pv^ON^ proprioceptors (Hippenmeyer et al., 2005). (D) Approximately 70% of Pv^ON^ (red) proprioceptive sensory neurons express βgal (green) in p7 *NB2::tauLacZ* mice (n = 3 L4 DRG, three mice). Scale bar represents 100 μm. (E) In situ hybridization against *NB2* reveals no expression in motor neurons. In *NB2::tauLacZ* mice, ChAT^ON^ (red) motor neurons in the ventral spinal cord do not express βgal (green; shown in inset). Scale bar represents 100 μm. (F) In the mouse spinal cord, Pv^ON^ proprioceptive sensory neurons form monosynaptic contacts onto motor neurons. GABApre interneurons modulate sensory input onto motor neurons through presynaptic inhibition of sensory afferent terminals. NB2 is expressed in a subset of proprioceptive (prop, Pv^ON^) and nonproprioceptive (nonprop, Pv^OFF^) sensory neurons. Venn diagram shows fraction of Pv^ON^/NB2^ON^ neurons as a function of Pv^ON^/NB2^OFF^ (prop) and Pv^OFF^/NB2^ON^ (nonprop) cells. See also Figure S1 and Table S1.

We found that five of the six contactins, *Cntn1*, *TAG-1* (*Cntn2*), *BIG-1* (*Cntn3*), *BIG-2* (*Cntn4*), and *NB2* (*Cntn5*) (Furley et al., 1990, Gennarini et al., 1989, Ogawa et al., 1996, Yoshihara et al., 1994, Yoshihara et al., 1995) were expressed by DRG neurons (Figure 1A; Table S1). Of these, *Cntn1*, *TAG-1*, and *BIG-2*, were also expressed by motor neurons and, based on our design constraints were therefore excluded from further analysis (Table S1). We failed to detect overlap in *BIG-1* and *Pv* expression (data not shown), whereas *Pv* exhibited extensive overlap with NB2 transcript and protein (Figures 1B–1C′). In addition, analysis of βgal expression in *NB2::tauLacZ* mice (Li et al., 2003) revealed overlap in βgal expression and Pv-positive (Pv^ON^) proprioceptors, as well as expression in a subset of Pv-negative (Pv^OFF^) sensory neurons (Figures 1D and 1F). In spinal cord, we found that neither endogenous *NB2*, nor βgal were expressed by motor neurons, marked by choline acetyltransferase (ChAT) expression (Figure 1E; for full spinal cord views and *NB2* probe specificity see Figures S1A–S1D). These data establish that NB2 is expressed by proprioceptive sensory but not motor neurons.

### Sensory NB2 Expression Ensures a High Density of GABApre Bouton Contacts

**Figure 2:**
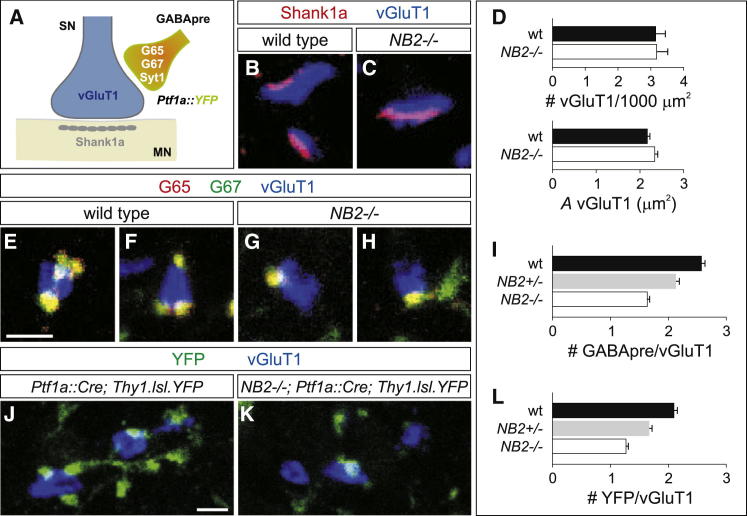
NB2 Promotes High-Density GABApre Bouton Contacts (A) Sensory neuron (SN) terminals express vGluT1 and are apposed to Shank1a on motor neurons (MN). GABApre boutons express GAD65 (G65), GAD67 (G67) and Syt1, and can be genetically labeled using mice in which *Ptf1a::Cre* drives expression of *Thy1.lsl.YFP* (abbreviated as *Ptf1a::YFP*). (B and C) vGluT1^ON^ (blue) sensory terminals are apposed to Shank1a (red) in the ventral spinal cord of p21 wild-type (B) and *NB2* mutant mice (C). (D) Similar number of vGluT1^ON^ sensory terminals per 1,000 μm^2^ area in the ventral spinal cord of p21 *NB2* mutants as compared to wild-type mice (wild-type: 3.14 ± 0.29, three mice; *NB2*^*−/−*^: 3.17 ± 0.34, three mice; t test, p = 0.95). Also, the size (*A*) of vGluT1^ON^ sensory terminals in wild-type and *NB2* mutant mice does not change (wild-type: 2.17 ± 0.06 μm^2^, n = 327 terminals, three mice; *NB2*^*−/−*^: 2.34 ± 0.07 μm^2^, n = 320 terminals, three mice; t test, p = 0.06). (E–H) Fewer G65^ON^ (red)/G67^ON^ (green) GABApre boutons contact vGluT1^ON^ (blue) sensory afferent terminals in p21 *NB*2 mutants (G and H) as compared to wild-type mice (E and F). Scale bar represents 2 μm. (I) Compiled average number of G65^ON^/G67^ON^ and Syt1^ON^/G67^ON^ GABApre boutons on vGluT1^ON^ sensory terminals is reduced by 17% and 36% in *NB2* heterozygous and homozygous mutant mice respectively (wild-type: 2.57 ± 0.06, n = 769 boutons, four mice; *NB2*^*+/−*^: 2.13 ± 0.06, n = 796 boutons, four mice; *NB2*^*−/−*^: 1.64 ± 0.04, n = 1193 boutons, six mice; ANOVA, p < 0.0001). (J and K) YFP^ON^ (green) GABApre terminals on vGluT1^ON^ (blue) sensory afferent terminals in *Ptf1a::Cre; Thy1.lsl.YFP* (J) and *NB2*^*−/−*^*; Ptf1a::Cre; Thy1.lsl.YFP* mice (K). In *Ptf1a::Cre; Thy1.lsl.YFP* mice, YFP is expressed in GABApre interneurons (Betley et al., 2009). Scale bar represents 2 μm. (L) Twenty-one percent and 40% fewer YFP^ON^ terminals form in *NB2*^*+/−*^*; Ptf1a::Cre; Thy1.lsl.YFP* and *NB2*^*−/−*^*; Ptf1a::Cre; Thy1.lsl.YFP* mice, respectively (wild-type: 2.10 ± 0.06, n = 840 boutons, three mice; *NB2*^*+/−*^: 1.67 ± 0.06, n = 886 boutons, three mice; *NB2*^*−/−*^: 1.27 ± 0.04, n = 1,161 boutons, four mice; ANOVA, p < 0.0001). All data reported as mean ± SEM. See also Figure S2.

To test the involvement of NB2 in the formation of GABApre-sensory contacts, we assessed synaptic organization in *NB2* heterozygous and homozygous null mutant mice (Li et al., 2003). *NB2* mutants survive, breed normally, and did not exhibit obvious locomotor abnormalities (Li et al., 2003). The number of Pv^ON^ proprioceptive neurons in lumbar DRG was similar in wild-type and *NB2* mutant mice analyzed at p7 (Figures S2A and S2B; data not shown). Moreover, density of proprioceptive sensory axons and the number and size of vGluT1^ON^ proprioceptive sensory terminals in the ventrolateral spinal cord, as well as their alignment with postsynaptic Shank1a protein expression was similar in p21 wild-type and *NB2* null mice (Figures 2A–2D; data not shown) (Betley et al., 2009). Thus, the differentiation of sensory-motor synapses appears unaffected by the loss of NB2 function.

We next examined whether NB2 expression is required for the organization of GABApre boutons on sensory terminals. For this analysis, we monitored the expression of two selective GABApre bouton markers, the GABA-synthetic enzyme GAD65, and the vesicle-associated protein Synaptotagmin1 (Syt1), both in the context of expression of the general GABAergic inhibitory marker GAD67 (Figure 2A) (Betley et al., 2009). Thus, the coincident expression of GAD65 or Syt1 with GAD67 provides a secure molecular definition of GABApre boutons.

In the ventral spinal cord of p21 *NB2* mutant mice we detected a 36% reduction in the number of GAD65^ON^/GAD67^ON^ boutons in contact with vGluT1^ON^ sensory terminals (ANOVA, p < 0.0001) (Figures 2E–2I) and a 37% reduction in the number of sensory-associated Syt1^ON^/GAD67^ON^ boutons (ANOVA, p < 0.0001) (Figure 2I). We did not observe an increase in the number of sensory terminal-associated GAD67^ON^ boutons that expressed either GAD65, or Syt1 alone, indicating there is a coordinate loss of these two defining GABApre bouton markers (data not shown). In addition, in *NB2* heterozygous mice we detected a 17% decrease in the number of GAD65^ON^/GAD67^ON^ and Syt1^ON^/GAD67^ON^ sensory-associated boutons (ANOVA, p < 0.0001) (Figure 2I), implying a dosage-dependence on NB2 expression level. Thus, sensory neuron expression of NB2 is required for the expression of defining GABApre bouton markers.

We next examined whether the coordinate loss of GABApre synaptic markers actually signifies the absence of GABApre boutons themselves. To assess this issue, we took advantage of the fact that GABApre neurons can be marked by lineage tracing on the basis of the Ptf1a transcriptional character of their progenitors (Betley et al., 2009, Glasgow et al., 2005). *Ptf1a::Cre; Thy1.lsl.YFP*-directed fluorescent protein (YFP) (Buffelli et al., 2003, Kawaguchi et al., 2002) was expressed in ∼70% of GAD65^ON^ GABApre terminals and was largely excluded from GABApost terminals that form contacts on motor neurons (Betley et al., 2009). The detection of some YFP^OFF^/GAD65^ON^ GABApre boutons is likely a consequence of the mosaic nature of reporter expression driven by the *Thy1.lsl.YFP* allele (Betley et al., 2009). In mice marked by *Ptf1a::Cre; Thy1.lsl.YFP* expression, we found that the number of YFP^ON^ GABApre boutons in contact with vGluT1^ON^ sensory terminals was reduced by 40% in *NB2* null mutants and by 21% in heterozygous *NB2* mice (ANOVA, p < 0.0001) (Figures 2J–2L). Thus, the absence of GAD65 and Syt1 in *NB2* mutant mice appears to reflect the loss of GABApre boutons from sensory terminals and not simply the absence of marker expression.

We also determined whether the reduction in GABApre bouton density on sensory terminals in *NB2* mutants is accompanied by the appearance of ectopic contacts on nonsensory targets. The synaptic localization of GAD65 is dependent on local sensory terminal-derived BDNF signaling (Betley et al., 2009), leading us to monitor the impact of *NB2* inactivation on the expression of the other defining GABApre marker, Syt1, in YFP^ON^ boutons. In p21 wild-type mice, we found that 91% of YFP^ON^/Syt1^ON^ boutons were associated with vGluT1^ON^ sensory terminals. We detected a similarly high incidence of YFP^ON^/Syt1^ON^ boutons associated with sensory terminals in *NB2* mutants (data not shown). We suspect that the few YFP^ON^/Syt1^ON^ processes that are separated from vGluT1^ON^ sensory terminals reflect a degree of vesicle accumulation in interterminal axonal domains. Together, these data support the idea that the loss of GABApre boutons from sensory terminals is not accompanied by the appearance of additional GABApre synapses with other neuronal targets, suggesting that sensory NB2 acts to promote the early elaboration of presynaptic boutons.

**Figure 3:**
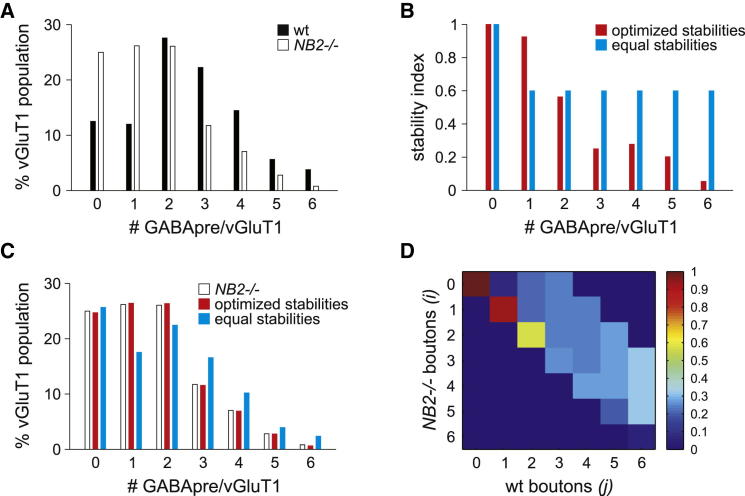
Enhanced Impact of NB2 Loss on High-Density GABApre-Sensory Synapses (A) Wild-type (black) and *NB2* mutant (white) GABApre bouton density frequency histograms. Bars represent the percent of vGluT1^ON^ sensory afferent terminals that receive zero to six GABApre boutons in wild-type and *NB2* mutant mice. (B) Probabilities of GABApre bouton maintenance in the *NB2* mutant using a quantitative model. In the equal stabilities model (blue), the probability of GABApre bouton maintenance was held constant at 0.6. In the optimized stabilities model (red), these probabilities were adjusted with respect to the experimentally observed GABApre bouton density distribution for the *NB2* mutant, revealing lower probabilities of GABApre bouton maintenance among high bouton-density GABApre synapses (see Supplemental Experimental Procedures). (C) GABApre bouton density distributions for *NB2* mutant mice (white), and equal (blue) and optimized (red) probabilities of GABApre bouton maintenance (see Supplemental Experimental Procedures). Applying equal stabilities across all GABApre synapses resulted in an overestimation of high bouton-density GABApre synapses. Optimal fitting of the experimental data required the reduction of GABApre bouton maintenance among high bouton-density GABApre synapses. (D) Optimized transition matrix for the *NB2* mutant. Each element (*i*, *j*) of the matrix is the probability of having *i* GABApre boutons on a sensory afferent terminal in the *NB2* mutant given *j* GABApre boutons in wild-type mice (see Supplemental Experimental Procedures).

We next considered whether the decrease in GABApre bouton packing density is spread evenly over the entire population of proprioceptive terminals, or reflects a preferential depletion from a smaller subset. Strikingly, in wild-type mice, the number of GABApre boutons in contact with individual sensory terminals varied from zero to ten, with a mean density of approximately three boutons/sensory terminal (Figure 3A) (Betley et al., 2009). In *NB2* mutants, we observed a clear reduction in the incidence of sensory terminals that possessed three or more GABApre boutons and in addition observed a doubling in the number of sensory terminals that lacked any associated GABApre boutons (Figure 3A). These observations suggest that inhibitory boutons are lost from sensory terminals that receive inputs across the spectrum of GABApre bouton packing densities.

We also examined whether the impact of NB2 varies as a function of GABApre bouton density. In *NB2* mutant mice, we observed a disproportionally large reduction in GABApre bouton number at the high end of the wild-type distribution range (those with four to six boutons/sensory terminal) (Figure 3A). To provide further insight into the question of whether high-density bouton arrangements are more sensitive to the loss of NB2, we modeled the impact of a uniformly applied 40% decrease in GABApre bouton number, comparing predicted and experimentally-derived bouton packing data (see Supplemental Experimental Procedures). We found that with the assumption of a uniformly-applied bouton loss, independent of starting packing density, there was a marked overestimation in the number of retained high bouton-density GABApre synapses in *NB2* mutants (Figures 3B and 3C). In contrast, a quantitative model that optimized the likelihood of retaining GABApre boutons in *NB2* mutants revealed that high bouton-density GABApre synapses are more vulnerable to the loss of NB2 (Figures 3B–3D). These modeling studies support the view that NB2 loss disproportionally strips GABApre bouton synapses from sensory terminals that exhibit a high bouton-packing density (Figure 6B).

### Sensory Caspr4 Serves as an NB2 Coreceptor for GABApre Boutons

**Figure 4:**
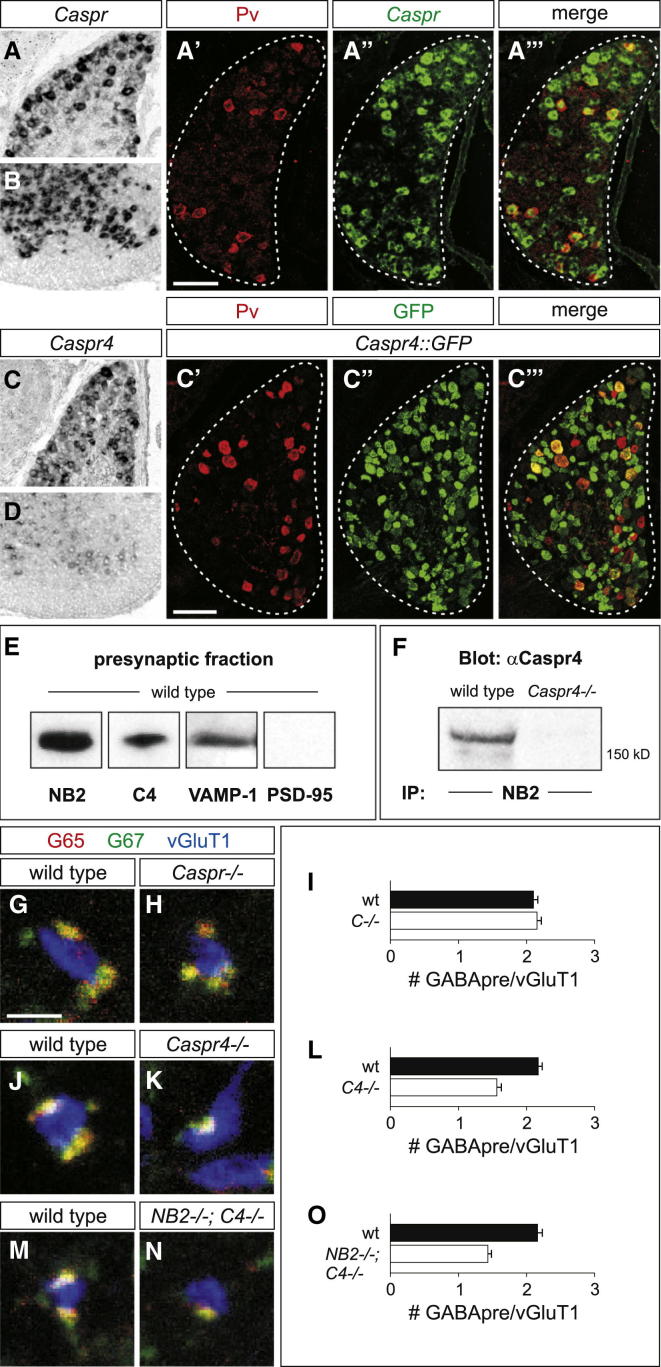
Caspr4 in Proprioceptive Sensory Neurons Serves as an NB2 Coreceptor (A and B) *Caspr* expression in p5 DRG (A) and motor neurons (B). Pv^ON^ (red) proprioceptive DRG neurons express *Caspr* (green) at p6 (A′–A″′). Scale bar represents 100 μm. (C and D) *Caspr4* is expressed at high levels in p5 DRG (C), but only weakly present in motor neurons (D). Fifty-three percent of Pv^ON^ (red) proprioceptive DRG neurons express GFP (green) in p7 *Caspr4::GFP* mice (C–C″′; n = 3 L4 DRG, three mice). Scale bar represents 100 μm. (E) Presynaptic preparations were made from p6 to p7 mouse spinal cords. The presynaptic protein, VAMP-1, a synaptic vesicle-associated protein, is expressed in this fraction, whereas the postsynaptic protein, PSD-95 is excluded. Using this preparation, we found that NB2 and Caspr4 are expressed presynaptically in the mouse spinal cord. Molecular weights are as follows: Caspr4 = 190 kDa; NB2 = 130 kDa; PSD-95 = ∼100 kDa; VAMP-1 = 17 kDa. (F) Brain lysates from wild-type and *Caspr4* mutant mice were immunoprecipitated using an NB2 antibody. Blotting with Caspr4 revealed an interaction between Caspr4 and NB2 that was absent in *Caspr4* mutant brains. (G–I) Normal numbers of G65^ON^ (red)/G67^ON^ (green) and Syt1^ON^/G67^ON^ boutons per vGluT1^ON^ (blue) sensory terminals in p21 wild-type (G) and *Caspr* mutant mice (H and I; wild-type: 2.10 ± 0.07, n = 563 boutons, three mice; *Caspr*^*−/−*^: 2.15 ± 0.06, n = 541 boutons, three mice; t test, p = 0.56). Scale bar represents 2 μm in (G), (H), (J), (K), (M), and (N). (J and K) A 28% reduction (mean) in number of G65^ON^ (red)/G67^ON^ (green) and Syt1^ON^/G67^ON^ GABApre boutons per vGluT1^ON^ (blue) sensory terminals in *Caspr4* mutants (K) as compared to wild-type mice (J and L; wild-type: 2.17 ± 0.06, n = 750 boutons, three mice; *Caspr4*^*−/−*^: 1.56 ± 0.07, n = 569 boutons, three mice; t test, p < 0.0001). Similar results were obtained for a second *Caspr4* mutant mouse line (data not shown). (M–O) A 34% reduction (mean) in the number of G65^ON^ (red)/G67^ON^ (green) and Syt1^ON^/G67^ON^ GABApre boutons per vGluT1^ON^ (blue) sensory terminals in *NB2; Caspr4* double mutant mice (N) as compared to wild-type mice (M and O; wild-type: 2.16 ± 0.07, n = 367 boutons, two mice; *NB2*^*−/−*^*; Caspr4*^*−/−*^: 1.43 ± 0.05, n = 534 boutons, three mice; t test, p < 0.0001). All data reported as mean ± SEM. See also Figure S3.

In nodes of Ranvier, the specialized localization of ion channels depends on the interaction of contactin proteins with transmembrane Caspr coreceptors (Poliak and Peles, 2003). We therefore analyzed whether Caspr proteins might function together with NB2 in the assembly of GABApre synapses on sensory terminals. Analysis of the expression of the five Caspr genes, *Caspr* (*Cntnap1*) to *Caspr5* (*Cntnap5*) (Peles et al., 1997, Poliak et al., 2003, Spiegel et al., 2002), in p5 to p7 DRG and spinal cord revealed that *Caspr*, *Caspr2*, and *Caspr4* were expressed by proprioceptive sensory neurons (Figures 4A–4A″′ and 4C–4C″′; data not shown). Moreover, in *NB2::tauLacZ*; *Caspr4::GFP* mice, we detected overlap of GFP and βgal in numerous Pv^ON^ sensory neurons (Figures S3M–S3R), suggesting that many proprioceptive sensory neurons express both Caspr4 and NB2. *Caspr* and *Caspr2* were also expressed at high levels by motor neurons, whereas *Caspr4* was expressed at much lower levels in motor neurons (Figures 4B and 4D; for full spinal cord views of *Caspr* and *Caspr4* as well as *Caspr4* probe specificity see Figures S3A–S3C; data not shown).

We attempted to localize NB2 and Caspr4 protein expression at sensory-motor synapses in the ventral spinal cord. Analysis of aldehyde, ethanol, and methanol-fixed and fresh-frozen sections of p6 and p21 spinal cords, however, failed to reveal NB2/Caspr4 immunoreactivity at sensory afferent terminals, even under conditions of antigen retrieval. To address synaptic localization of NB2 and Caspr4 biochemically, we isolated the presynaptic fraction of synaptosomal preparations from p6 to p7 spinal cord (Phillips et al., 2001). As controls, we detected the presynaptic protein marker, VAMP-1 but not the postsynaptic protein marker PSD-95 in such preparations (Figure 4E). In addition, we detected NB2 and Caspr4 protein expression in this presynaptic fraction (Figure 4E), providing biochemical evidence that both proteins are expressed in nerve terminals in the postnatal spinal cord. One potential explanation for the lack of synaptic protein immunoreactivity in histological sections is that NB2 and Caspr4 form a protein complex in which the antigen epitope is masked or otherwise occluded (Fritschy et al., 1998).

We next determined whether Caspr4 interacts with NB2 in brain tissue. We probed the interaction of Caspr4 with NB2 in brain lysates by immunoprecipitation, revealing that Caspr4 forms a complex with NB2 in wild-type, but not in *Caspr4* mutant, brain tissue (Figure 4F). In addition, Caspr4 and NB2 associate in transfected cells in vitro (Figures S3S and S3T). Together, these data provide evidence that NB2 and Caspr4 form an interaction complex in neural tissue.

Based on these findings, we explored whether loss of Caspr4, like that of NB2, prevents high bouton-packing. We found that the overall trajectory of Pv^ON^ proprioceptive axons was similar in p7 *Caspr4* mutants as compared to wild-type mice (Figures S3D and S3E). In addition, the number and size of vGluT1^ON^ sensory terminals, as well as their alignment with motor neuron Shank1a plaques was similar in wild-type and *Caspr4* mutant mice (Figures S3F–S3K). Analysis of p21 *Caspr4* mutants revealed that the number of GAD65^ON^/GAD67^ON^ GABApre boutons on sensory terminals was reduced by 39% (t test, p < 0.0001) (Figures 4J–4L and S3L). In contrast, analysis of *Caspr* (Gollan et al., 2003) and *Caspr2* (Poliak et al., 2003) mutants revealed no change in the density of GABApre boutons (Figures 4G–4I; data not shown), indicating the specificity of Caspr4 function.

If both NB2 and Caspr4 act in the same pathway, we might anticipate a similar severity of phenotype when ablating one or both components of this putative receptor complex. We therefore analyzed GABApre bouton formation in mice in which both *NB2* and *Caspr4* genes were inactivated. In *NB2; Caspr4* double mutant mice, we detected a 34% reduction in GAD65^ON^/GAD67^ON^ and Syt1^ON^/GAD67^ON^ GABApre boutons in contact with vGluT1^ON^ sensory terminals (t test, p < 0.0001) (Figures 4M–4O), a reduction similar in extent to that observed in *NB2* and *Caspr4* single mutant mice. These genetic data support the view that NB2 and Caspr4 act as coreceptors on sensory terminals to direct the formation of GABApre bouton synapses.

### A Role for L1 Ig Proteins in GABApre Recognition of Sensory Terminals

Certain Schwann cell-axonal interactions in myelinated nerves are mediated by binding of contactins to various members of the L1 Ig superfamily (Poliak and Peles, 2003, Shimoda and Watanabe, 2009). We therefore examined whether any of the four L1 family members—L1, close homolog of L1 (CHL1), neurofascin (NF), and NrCAM—might function in GABApre neurons as ligands for sensory terminal-expressed NB2/Caspr4.

**Figure 5:**
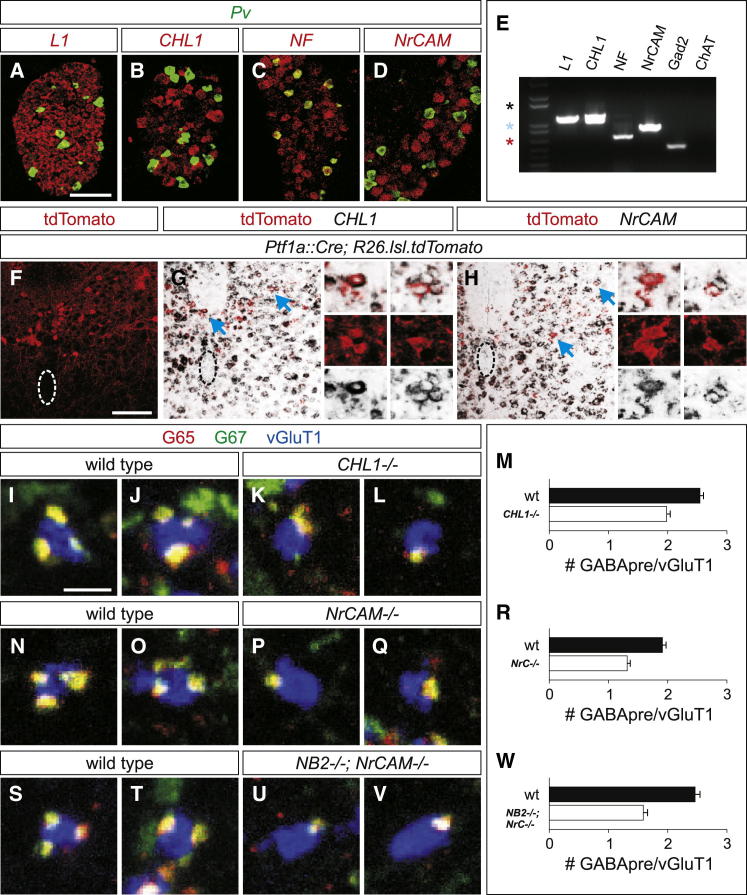
A Role for L1 Family Members in GABApre Recognition of Sensory Terminals (A–D) Assessing mRNA expression of L1 family members in p6 DRG using double fluorescent in situ hybridization. *Pv*^*ON*^ (green) proprioceptive sensory neurons express *NF* (red, C). *Pv* does not colabel with *L1* (red, A), *CHL1* (red, B), or *NrCAM* (red, D). Based on its expression in proprioceptive sensory neurons, we excluded NF as a *trans*-acting candidate for NB2/Caspr4. Scale bar represents 100 μm. (E) RT-PCR of L1 family member transcripts using p0 *Ptf1a::Cre; Rosa26.lsl.YFP* cDNA. cDNA was generated from FACS-sorted YFP^ON^ cells from *Ptf1a::Cre; Rosa26.lsl.YFP* lumbar spinal cords. *Gad2* (*GAD65*) serves as a positive control because it is known to be expressed in YFP^ON^ neurons of *Ptf1a::Cre; Thy1.lsl.YFP* mice (Betley et al., 2009). *ChAT* is not expressed in Ptf1a-derived YFP^ON^ interneurons and thus serves as a negative control. Stars: 1,600 bp (black); 1,000 bp (blue); 650 bp (red). (F) tdTomato^ON^ (red) cells in the intermediate spinal cord of p7 *Ptf1a::Cre; Rosa26.lsl.tdTomato* mice (dotted outline indicates central canal). Scale bar represents 100 μm. (G and H) *CHL1* (G) and *NrCAM* (H) coexpression with tdTomato^ON^ neurons in the intermediate zone of p7 *Ptf1a::Cre; Rosa26.lsl.tdTomato* spinal cords (dotted outline indicates central canal). Small images show magnifications of individual colabeled neurons indicated with blue arrows. (I–M) Mean number of GABApre synapses is reduced by 22% in *CHL1* mutants (K–M) as compared to wild-type mice (I, J, and M; wild-type: 2.54 ± 0.06, n = 579 boutons, three mice; *CHL1*^*−/−*^: 1.98 ± 0.06, n = 538 boutons, three mice; t test, p < 0.0001). Scale bar represents 2 μm in (I)-(L), (N)-(Q), and (S)-(V). (N–R) vGluT1^ON^ (blue) sensory terminals in *NrCAM* mutant mice (P–R) receive 31% fewer GAD65 (G65)^ON^ (red)/GAD67 (G67)^ON^ (green) and Syt1^ON^/G67^ON^ GABApre boutons as compared to wild-type mice (N, O, and R; wild-type: 1.91 ± 0.06, n = 522 boutons, three mice; *NrCAM*^*−/−*^: 1.31 ± 0.05, n = 538 boutons, three mice; t test, p < 0.0001). (S–W) Analysis of *NB2*^*−/−*^*; NrCAM*^*−/−*^ double mutants (U–W) showed a mean 36% reduction in the number of G65^ON^ (red)/G67^ON^ (green) and Syt1^ON^/GAD67^ON^ GABApre boutons located on vGluT1^ON^ (blue) sensory terminals as compared to wild-type mice (S, T, and W; wild-type: 2.46 ± 0.08, n = 362 boutons, two mice; *NB2*^*−/−*^*; NrCAM*^*−/−*^: 1.59 ± 0.07, n = 379 boutons, two mice; t test, p < 0.0001). All data reported as mean ± SEM. See also Figure S4.

We monitored expression of *L1*, *CHL1*, *NF*, and *NrCAM* in wild-type p5 to p7 DRG and spinal cord and found broad transcript expression by many spinal cord and DRG neurons (Figures 5A–5D; for full spinal cord views see Figures S4A–S4D). In situ hybridization histochemistry revealed that neither *L1*, *CHL1*, nor *NrCAM* were expressed by *Pv*^*ON*^ proprioceptive sensory neurons (Figures 5A, 5B and 5D). To assess expression of L1 members in Ptf1a-derived dI4 interneurons that include the GABApre set we FACS-isolated YFP^ON^ interneurons from the spinal cord of p0 *Ptf1a::Cre; Rosa26.lsl.YFP* mice (Betley et al., 2009, Srinivas et al., 2001). All four L1 family transcripts were expressed by YFP^ON^ neurons, as detected in a RT-PCR analysis (Figure 5E). Moreover, we detected *NrCAM* and *CHL1* expression in tdTomato^ON^ cells in the intermediate region of *Ptf1a::Cre; Rosa26.lsl.tdTomato* mice (Figures 5F–5H and S4E), the assigned location of GABApre neuronal cell bodies (Betley et al., 2009, Hughes et al., 2005).

To assess the functional role of L1 family members, we analyzed GABApre bouton contacts with sensory terminals in *L1*, *CHL1*, and *NrCAM* mutant mice (Dahme et al., 1997, Montag-Sallaz et al., 2002, Sakurai et al., 2001). The organization of proprioceptive sensory terminals was similar in wild-type, *L1*, *CHL1*, and *NrCAM* mutants (Figures S4H, S4I, S4L, S4M, and S4S). Moreover, in these three L1 family mutants, vGluT1^ON^ sensory terminals on motor neurons remained juxtaposed to the postsynaptic motor neuron marker Shank1a (Figures S4F, S4G, S4J, and S4K). Analysis of the density of GABApre boutons on vGluT1^ON^ sensory terminals revealed a 31% reduction in *NrCAM* mutants (t test, p < 0.0001) (Figures 5N–5R and S4N), a 22% reduction in *CHL1* mutants (t test, p < 0.0001) (Figures 5I–5M), but no change in *L1* mutants (t test, p = 0.65) (Figures S4O–S4R and S4T). Thus, elimination of NrCAM and CHL1 from GABApre interneurons decreases GABApre bouton density on sensory terminals, consistent with a role for these two L1 family members in the organization of GABApre synapses.

**Figure 6:**
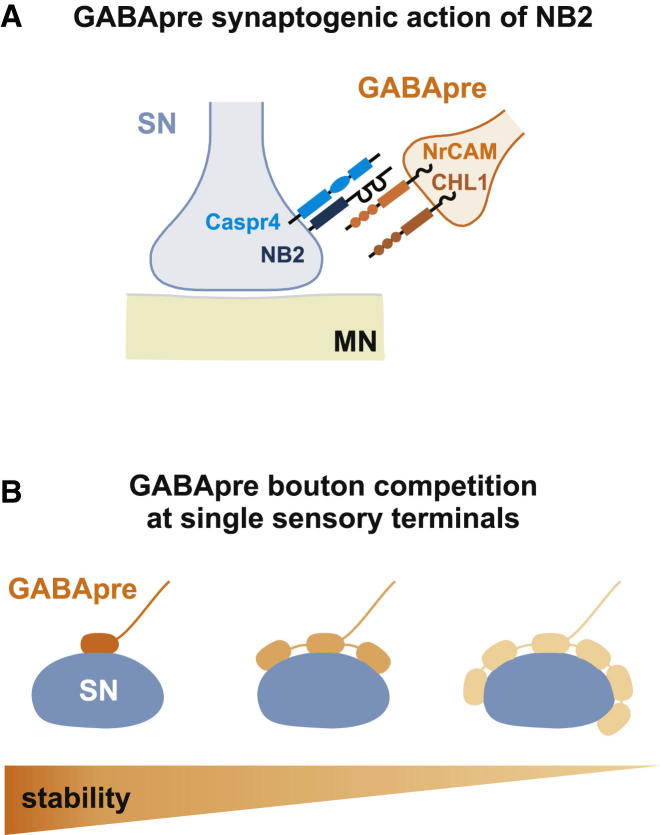
An NB2/Caspr4 Adhesion Complex Regulates GABApre Synaptic Organization (A) Sensory (SN)-derived NB2 and Caspr4 act together with GABApre-derived NrCAM and CHL1 as an adhesive complex in the formation of GABApre-sensory terminals. (B) GABApre boutons compete for sensory terminal space. Sensory neuron (SN) terminals with larger GABApre bouton density show lower GABApre stability compared to sensory terminals with few GABApre boutons. Fading color of GABApre boutons indicates relative stability of individual GABApre boutons on sensory terminals.

To ask whether NrCAM functions in the same recognition process as NB2, we generated mice in which both NB2 and NrCAM were deleted. *NB2; NrCAM* double mutant analysis showed a 36% reduction in the number of GABApre boutons that form on vGluT1^ON^ sensory terminals (t test, p < 0.0001) (Figures 5S–5W). This observation supports the idea that GABApre-derived NrCAM and sensory-derived NB2 form a ligand-receptor pair that directs high-density GABApre bouton formation with sensory afferent terminals (Figure 6A).

## Discussion

Axoaxonic synapses are a specialized feature of primary sensory circuits in the mammalian CNS, providing a structural substrate for the selective filtering of afferent information. Moreover, inactivation of GABApre neurons in mice results in a profound disruption in skilled motor behavior (A. Fink and T.M.J., personal communication), emphasizing the specialized developmental, anatomical, and functional properties of this inhibitory synapse. Our analysis of the developmental organization of presynaptic contacts with proprioceptive sensory terminals indicates that the formation of this axoaxonic arrangement has its basis in an immunoglobulin recognition complex mediated, in part, by interactions between sensory NB2/Caspr4 and interneuron NrCAM/CHL1 that promote or stabilize contacts between the axons of GABApre interneurons and the terminals of sensory afferents (Figure 6A). Restricted expression of the L1 family protein NF underlies the focal accumulation of GABAergic synapses at the axon initial segment of Purkinje neurons (Ango et al., 2004), suggesting a more general function of L1 family proteins in synaptic organization. Thus neuronal Ig proteins may have conserved roles in defining spatial domains of synaptogenesis in the mammalian CNS, in addition to their well-established functions in defining specific membrane domains at and around the nodes of Ranvier (Feinberg et al., 2010, Gollan et al., 2003, Poliak et al., 2003, Sherman et al., 2005, Traka et al., 2003).

Inactivation of NB2/Caspr4 and CHL1/NrCAM proteins (either as single mutants or in combination as double mutants) elicits only a partial reduction in the number of GABApre boutons on sensory terminals, indicating that other recognition systems function together with this set of Ig proteins. One plausible idea is that related Ig proteins serve overlapping functions in instructing presynaptic contacts on sensory terminals. Indeed, Cntn1 and TAG-1 are also expressed by proprioceptive sensory neurons, although the function of their known interacting partners, Caspr and Caspr2, is not required for GABApre bouton packing, at least when Caspr proteins are inactivated individually (Figure 4; data not shown). We note that NB2 is expressed in cutaneous sensory neurons in the DRG (Figure 1F), and thus could have a general role in mediating presynaptic inhibition onto other sensory afferents. Moreover, other recent studies have implicated contactins in synaptic assembly in the chick retina (Yamagata and Sanes, 2012), indicating a more general synaptogenic function for this set of recognition proteins.

Our quantitative studies are consistent with the idea that depletion of sensory terminal NB2 expression covaries with presynaptic packing density: sensory terminals with the greatest density of GABApre boutons appear most sensitive to loss of NB2. We speculate that GABApre boutons normally establish axoaxonic contacts with their target sensory terminals under conditions of competition. The rarity of axoaxonic synaptic arrangements characterized by higher numbers of GABApre boutons presumably reflects the limited availability of sensory terminal target membrane. In essence, our findings suggest the operation of a competitive program of GABApre bouton stabilization, exerted at the level of individual sensory terminals (Figure 6B).

In many regions of the CNS, inputs to individual neurons are pruned extensively through competitive mechanisms to achieve a final, functionally-appropriate, innervation density (Buffelli et al., 2003, Kwon et al., 2012). In the peripheral nervous system, the geometry of postsynaptic dendritic domains of ciliary ganglion neurons defines the number and spacing of their synaptic inputs (Hume and Purves, 1981). We observe a 10-fold variation in the density of GABApre bouton packing between individual sensory terminals, which may reflect functional heterogeneity in the local organization of presynaptic inhibitory circuits (Quevedo et al., 1997, Walmsley et al., 1987).

Our findings on the role of neuronal Ig proteins in establishing the packing density of inhibitory synapses complements and extends studies on the transcriptional control of inhibitory synapse number. Most notably, the transcription factor NPAS4 has been shown to regulate the density of inhibitory synapses in the mammalian CNS (Lin et al., 2008). Future studies may help to define how transcriptional mechanisms and Ig-based recognition conspire to establish the final density of inhibitory synapses in defined circuits within the mammalian CNS.

## Experimental Procedures

### Mouse Strains

The following mouse strains were used in this study (*lsl* designates a *loxP.STOP.loxP* cassette): *Caspr* (Gollan et al., 2003), *Caspr2* (Poliak et al., 2003), *Caspr4* (GFP knockin line where GFP-pA followed by PGK-Neo-pA is knocked in immediately following the methionine start codon in the *Caspr4* gene; T. Karayannis, E. Au, E. Peles and G. Fishell, personal communication; requests for this mutant should be addressed to E. Peles), *CHL1* (Montag-Sallaz et al., 2002), *Kirrel-3* (Prince et al., 2013), *L1* (Dahme et al., 1997), *NB2* (tauLacZ knockin line) (Li et al., 2003), *NrCAM* (Sakurai et al., 2001), *Ptf1a::Cre* (Kawaguchi et al., 2002), *Pv::Cre* (Hippenmeyer et al., 2005), *Rosa26.lsl.YFP* (Srinivas et al., 2001), *Rosa26.lsl.tdTomato* (Jackson, Ai14) (Madisen et al., 2010), and *Thy1.lsl.YFP* (line 15) (Buffelli et al., 2003). Experiments conform to the regulatory standards of the Institutional Animal Care and Use Committee of Memorial Sloan-Kettering Cancer Center.

### Candidate Screen

We identified genes coding for candidate receptors by searching the National Center for Biotechnology Information (NCBI) for transcripts in the mouse genome that were predicted to code an extracellular Ig domain and either a transmembrane domain and internal PDZ binding motif or a GPI anchor to the membrane. We performed in situ hybridization analysis on p5 to p6 mouse spinal cord and DRG tissue with probes designed to anneal to these transcripts. Candidates that showed high level of expression in sensory neurons and not motor neurons were further assessed for expression specifically in proprioceptive sensory neurons by performing double in situ hybridizations with the proprioceptive marker gene *Parvalbumin* (*Pv*).

### Histochemistry

In situ and double fluorescent in situ hybridization histochemistry on 12 μm thick cryostat sections was performed as described previously (Arber et al., 1999, Price et al., 2002). In situ hybridization histochemistry combined with antibody staining was performed as described in Ashrafi et al. (2012). tdTomato detection in combination with in situ hybridization was performed with additional TSA amplification (Perkin-Elmer) of the RFP antibody. Antisense and sense in situ probes were generated from mouse e12.5/p6 spinal cord, DRG, and brain cDNAs using PCR amplification. Probes ranged in length from ∼600 to 1,300 bp. *CHL1* antisense probe was generated from a full-length mouse clone (ThermoFisher MMM1013-211694136).

### Immunohistochemistry

Immunohistochemistry on 12 μm thick cryostat sections of lumbar level (L) 4 to 5 spinal cord was performed as previously described (Betley et al., 2009). Rabbit anti-βgal (gift from J. Sanes) (Gray and Sanes, 1991), rabbit anti-ChAT (generously provided by S.B.-M. and T.M.J., unpublished data), rabbit anti-GAD65 1:50,000 (Betley et al., 2009), mouse anti-GAD67 1:10,000 (Millipore), rabbit anti-GAD67 1:10,000 (Betley et al., 2009), chicken anti-GFP 1:1,000 (Millipore), sheep anti-GFP 1:1,000 (Molecular Probes), rat anti-NB2 (1A6) 1:4 (Shimoda et al., 2012), chicken anti-Pv 1:10,000 (generously provided by S.B.-M. and T.M.J., unpublished data), rabbit anti-RFP (Rockland), rabbit anti-Shank1a 1:64,000 (Betley et al., 2009), rabbit anti-Shank1a 1:1,000 (Millipore), mouse anti-Syt1 1:100 (ASV48, Developmental Studies Hybridoma Bank), and guinea pig anti-vGluT1 1:32,000 (Betley et al., 2009).

### Synaptic Quantification

Synaptic quantifications were performed using Leica LAS software plug-in (Version 2.3.1 build 5194) on z stacks (0.5 μm optical sections) obtained on a Leica TCS SP5 confocal. At least three animals per genotype were analyzed and ∼100 vGluT1^ON^ terminals were counted per animal. Differences between wild-type and mutant mice were assessed using t test (when comparing two groups) or ANOVA (when comparing three groups) (significant at p < 0.05). Data are reported as mean ± SEM.

### Quantitative Modeling

The probability of GABApre bouton maintenance on individual sensory afferent terminals was estimated using wild-type and *NB2* mutant GABApre-density data distributions. The underlying set of conditional probability mass functions was parameterized, and these parameters were interpreted as GABApre bouton synaptic stabilities in the context of loss of NB2. Parameters were optimized using a constrained linear least-squares approach (Supplemental Experimental Procedures).

### Synaptosomal Preparation and Immunoprecipitation

Synaptosomal membranes were prepared using Syn-Per (Thermofisher/Pierce) and pelleted at 15,000 × g for 20 min. The presynaptic fraction was isolated as described in Phillips et al. (2001). The nonsynaptic proteins were extracted from the pellet using a low pH buffer. The pellet was resuspended in Tris pH 8.0, 1% TX-100, 1 mM CaCl_2_, 1 mM MgCl_2_ and protease inhibitors, and incubated on ice for 20 min to extract the presynaptic proteins. The insoluble fraction was pelleted at 40, 000 × g for 20 min.

Expression plasmids containing Caspr family cDNAs were described previously (Peles et al., 1997, Poliak et al., 1999, Spiegel et al., 2002). NB2-myc cDNA was prepared by inserting a myc-tag sequence after the signal sequence of rat NB2 by PCR (the first 18 amino acids were removed and the sequence was ligated 54 bp from ATG codon). Transient expression in HEK293T cells, preparation of brain and cell lysates, immunoprecipitation, and western blot analysis was performed as described previously (Gollan et al., 2003).

The following antibodies were used for biochemical experiments: rat anti-NB2 (1A6) (Shimoda et al., 2012), rat anti-NB2 (1B10) (Toyoshima et al., 2009, Shimoda et al., 2012), rabbit anti-Caspr (Peles et al., 1997), rabbit anti-Caspr2 (Poliak et al., 1999), rabbit anti-Caspr3 (Spiegel et al., 2002), rabbit anti-Caspr4 (Spiegel et al., 2002), rabbit anti-Caspr5 (antibody was made by immunizing rabbits with a GST-fusion protein containing the intracellular domain of human Caspr5), mouse anti-myc (clone 9E10; Roche), rabbit anti-VAMP-1 (SYSY), and mouse anti-PSD-95 (Thermo Scientific).

### Fluorescence-Activated Cell Sorting and RT-PCR

To isolate putative GABApre neurons, YFP^ON^ cells from p0 *Ptf1a::Cre; Rosa26.lsl.YFP* mice were purified using fluorescence-activated cell sorting (FACS). Briefly, spinal cords were dissociated using Papain dissociation kit (Worthington) and sorted based on YFP fluorescence. RNA was then isolated using the Absolutely RNA Nanoprep Kit (Agilent) and cDNA was generated from these cells using WT-Pico Ovation Amplification Kit (NuGEN). RT-PCR was performed on cDNA generated from purified RNA using the following primers: ChAT (forward primer (FP): TCAGGGCAGCCTCTCTGTAT, reverse primer (RP): ATGTTGTCCACCCGACCTTC), CHL1 (FP: AGGACAGCGAAACTCTGGAA, RP: TCGTGTTCTGCATTTTGAGC), GAD2 (FP: AAAATCTCTTGGGCCCTTTC, RP: CCGGAGTCTCCATAGAGCAG), L1 (FP: CAAAGTCCAGGCAGTGAACA, RP: CTGTACTCGCCGAAGGTCTC), NF (FP: ACCTGGAGACCATCAACCTG, RP: TCAGGCAAGGGAATAGATGG), NrCAM (FP: AATCCAGTGTGAGGCCAAAG, RP: GAAAGCACGAGGTTTTGAGG).

## Author Contributions

S.A., J.N.B., J.D.C., S.B.-M., V.B., and J.A.K. performed experiments. S.A., J.N.B., J.D.C., E.P., T.M.J., and J.A.K. designed the study and interpreted results. E.P., S.B.-M., Y.S., and K.W. provided reagents. S.A., J.N.B., J.D.C., T.M.J., and J.A.K. wrote the paper.

## Supplemental Information

Document S1. Supplemental Experimental Procedures, Figures S1–S4, and Table S1
