# Supplementary material for: Neuronal Ig/Caspr Recognition Promotes the Formation of Axoaxonic Synapses in Mouse Spinal Cord
Source: Neuron. 2014 Jan 8;81(1):120–9. doi: 10.1016/j.neuron.2013.10.060 (PMC3898991; doi:10.1016/j.neuron.2013.10.060)
Supplement: Document S1. Supplemental Experimental Procedures, Figures S1–S4, and Table S1 [file mmc1.pdf]

Neuron, Volume *81*

Supplemental Information

Neuronal Ig/Caspr Recognition Promotes the Formation of Axoaxonic Synapses in Mouse Spinal Cord

Soha Ashrafi, J. Nicholas Betley, John D. Comer, Susan Brenner-Morton, Vered Bar, Yasushi Shimoda, Kazutada Watanabe, Elior Peles, Thomas M. Jessell, and Julia A. Kaltschmidt

SUPPPLEMENTAL INFORMATION

SUPPLEMENTAL FIGURES

Supplemental Figure 1

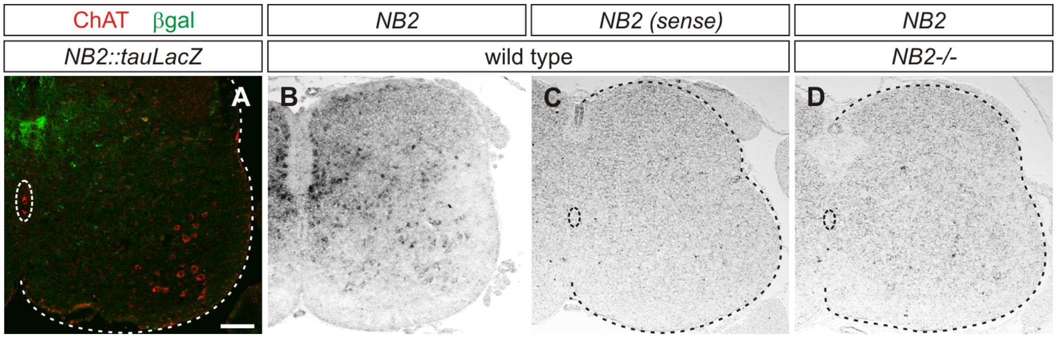

Supplemental Figure 1, related to Figure 1: Whole spinal cord views of NB2 expression

(A) *NB2::tauLacZ* p7 mice stained for βgal (green) and ChAT (red). Note absence of βgal expression in ChAT^{ON} motor neurons.

(B and C) Whole spinal cord views of expression of *NB2* anti-sense (B) and sense (C) probes at p6 to 7.

(D) No expression of *NB2* (anti-sense probe) on p7 *NB2* mutant spinal cord.

Scale bar: 100 μm in A.

Supplemental Figure 2

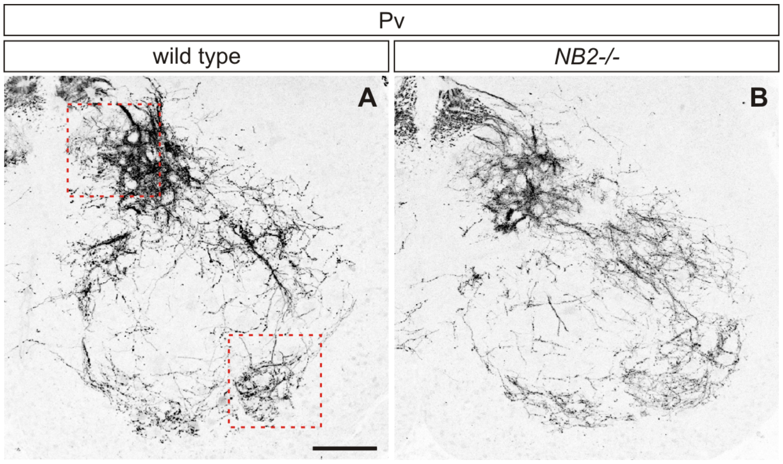

Supplemental Figure 2, related to Figure 2: Proprioceptive sensory axon guidance is maintained in *NB2* mutant mice

(A and B) Pv^{ON} (black) sensory projections into the spinal cord are normal in p7 *NB2* mutant mice (B) as compared to wild type mice (A). Measurement of Pv^{ON} pixel counts showed comparable levels of Pv expression between wild type and *NB2* mutant mice in the intermediate branching zone (upper red box; 3 mice per genotype; Mann-Whitney U test, p= 0.40) as well as in the ventral motor pool (lower red box; 3 mice per genotype; Mann-Whitney U test, p= 0.91).

Scale bar: 100 μm in A and B.

Supplemental Figure 3

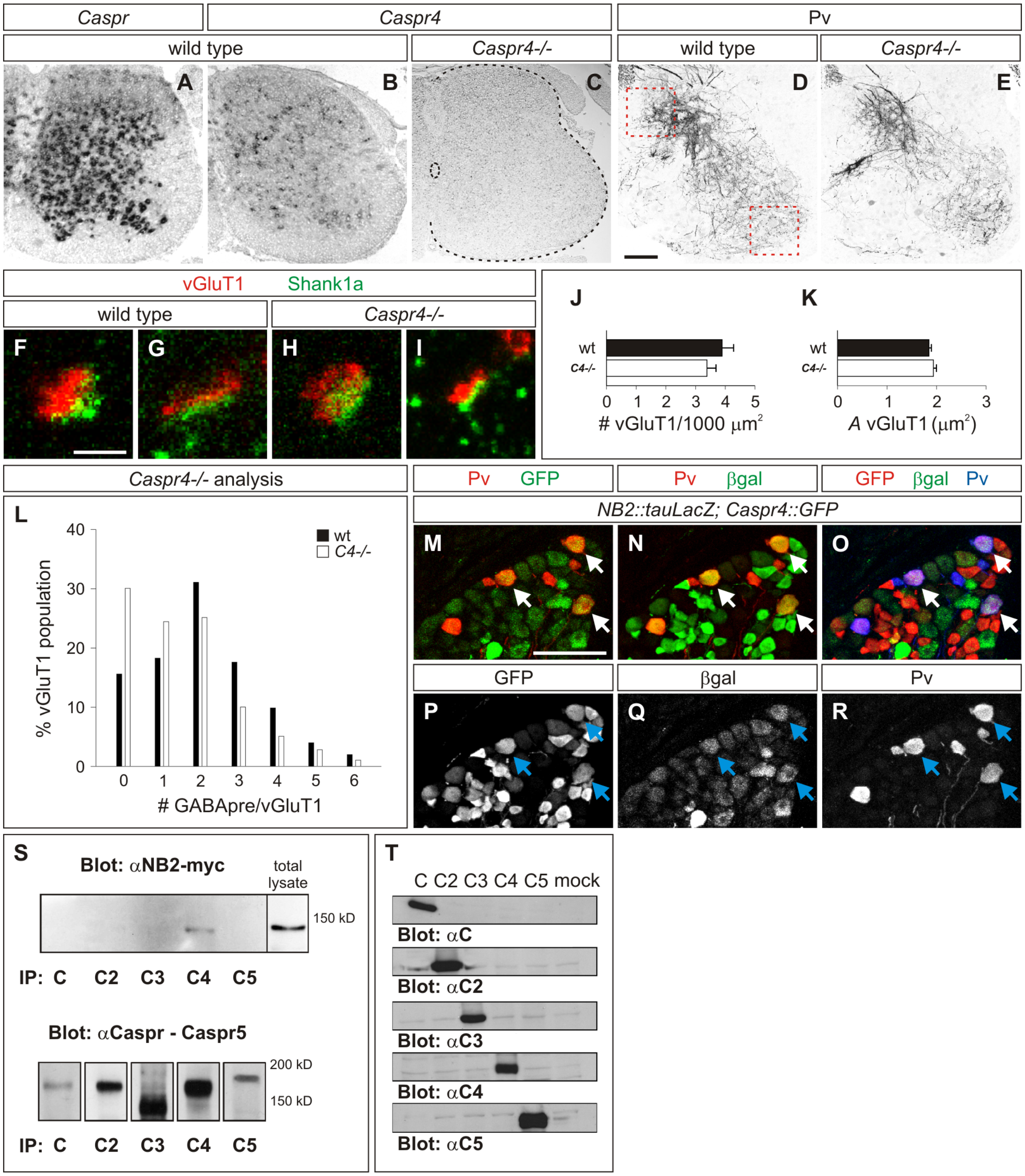

Supplemental Figure 3, related to Figure 4: *Caspr4* expression and interaction with NB2

(A) Expression of *Caspr* in p5 spinal cord.

(B and C) *Caspr4* expression in p5 wild type (B) and p7 *Caspr4* mutant spinal cords (C), showing specificity of the *Caspr4 in situ* probe.

(D and E) Normal Pv^{ON} (black) sensory innervation in wild type (D) and *Caspr4* mutant mice (E) at p7. Expression of Pv is similar between genotypes in the intermediate branching zone (upper red box; 3 mice per genotype; Mann-Whitney U test, p= 0.89) and ventral horn (lower red box; 3 mice per genotype; Mann-Whitney U test, p= 0.14) based on pixel count measurements.

(F-K) $vGluT1^{ON}$ (red) sensory terminals are aligned with Shank1a (green) in wild type (F and G) and *Caspr4* mutant mice (H and I). $vGluT1^{ON}$ sensory terminals in *Caspr4* mutants are present in normal numbers per 1000 μm^2 (J; wild type: 3.89 ± 0.38, 3 mice; $Caspr4^{-/-}$: 3.38 ± 0.31, 3 mice; t-test, p= 0.31) and have normal cross-sectional area (*A*) (K; wild type: 1.84 ± 0.05 μm^2, n = 328 terminals, 3 mice; $Caspr4^{-/-}$: 1.93 ± 0.06 μm^2, n = 314 terminals, 3 mice; t-test, p= 0.26).

(L) $vGluT1^{ON}$ sensory terminals in *Caspr4* mutants receive fewer $GAD65^{ON}/GAD67^{ON}$ and $GAD67^{ON}/Syt1^{ON}$ GABApre boutons. Similar to the *NB2* mutant, the population of sensory terminals with higher number of GABApre boutons is decreased.

(M-R) Mice heterozygous for both *NB2::tauLacZ* and *Caspr4::GFP* show expression of βgal and GFP in the same Pv^{ON} proprioceptive sensory neurons at p7 (white (M-O) and blue (P-R) arrows).

(S) NB2 specifically associates with Caspr4 in a co-immunoprecipitation assay. Lysates from HEK-293T cells co-transfected with myc-tagged NB2 and either Caspr - Caspr5 were subjected to immunoprecipitation using antibodies to each Caspr. Western blot analysis was carried out using an antibody to myc (top panel) and the corresponding

Caspr protein (bottom panel). Total represents a sample of the lysate used. The location of molecular mass markers is shown in kDa.

(T) Lysates from HEK-293T cells transfected with individual *Caspr* genes were blotted using antibodies to each Caspr protein. Expression of the protein by Western blot was only detected for the gene that was transfected, suggesting that the antibodies used are specific and do not cross-react with other Caspr proteins.

Scale bars: 100 μm in D, E and M-R; 2 μm in F-I. All data reported as mean ± SEM.

Supplemental Figure 4

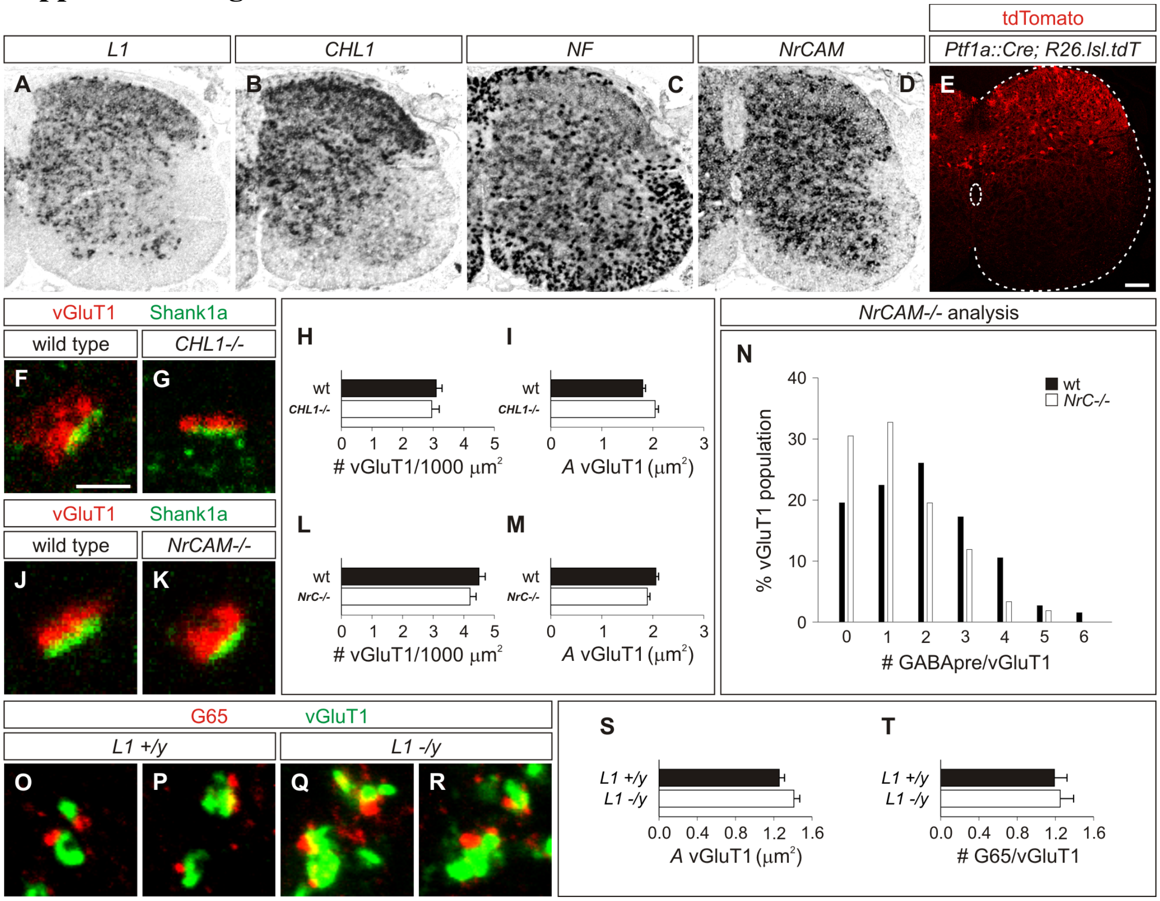

Supplemental Figure 4, related to Figure 5: L1 family spinal cord expression and normal sensory-motor synapses in *NrCAM*, *CHL1* and *L1* mutant mice

(A-D) *L1* (A), *CHL1* (B), *NF* (C) and *NrCAM* (D) transcript expression in p5 spinal cords.

(E) Spinal cord section from p7 *Ptf1a::Cre; Rosa26.lsl.tdTomato (R26.lsl.tdT)* mouse showing tdTomato expression in the intermediate zone and dorsal horn.

(F-I) vGlut1^{ON} (red) sensory terminals are aligned with Shank1a (green) in wild type (F) and *CHL1* mutant mice (G). While CHL1 deficient sensory synapses are on average 10% larger than wild type terminals (I; wild type: 1.80 ± 0.05 μm^2, n = 283 terminals, 3 mice; *CHL1*$^{-/-}$: 2.04 ± 0.07 μm^2, n = 286 terminals, 3 mice; t-test, p= 0.05), the average number

of sensory terminals per 1000 μm^2 area does not change (H; wild type: 3.09 ± 0.19, 3 mice; *CHL1*$^{-/-}$: 2.95 ± 0.25, 3 mice; t-test, p= 0.64).

(J-M) Normal alignment of vGluT1^{ON} (red) sensory terminals with Shank1a (green) in *NrCAM* mutants (K) compared to wild type mice (J). The area of vGluT1^{ON} sensory terminals is slightly decreased (8%) in *NrCAM* mutant mice (M; wild type: 2.06 ± 0.05 μm^2, n = 292 terminals, 3 mice; *NrCAM*$^{-/-}$: 1.89 ± 0.05 μm^2, n = 295 terminals, 3 mice; t-test, p= 0.02), however the average number per 1000 μm^2 area is unchanged (L; wild type: 4.49 ± 0.20, 3 mice; *NrCAM*$^{-/-}$: 4.21 ± 0.20, 3 mice; t-test, p= 0.31).

(N) vGluT1^{ON} sensory terminals in *NrCAM* mutants receive fewer GAD65^{ON}/GAD67^{ON} and GAD67^{ON}/Syt1^{ON} GABApre boutons. The population of sensory terminals with higher number of GABApre terminals is decreased.

(O-T) The average area of sensory terminals is unchanged in *L1* deficient mice as compared to *L1* hemizygous mice (S; *L1+/y*: 1.25 ± 0.06 μm^2, n = 80 boutons, 1 mouse; *L1-/y*: 1.41 ± 0.06 μm^2, n = 81 boutons, 1 mouse; t-test, p= 0.07). GAD65 (G65)^{ON} (red) GABApre terminals on vGluT1^{ON} (green) sensory terminals are present in similar numbers between *L1* deficient (Q, R and T) and *L1* hemizygous mice (O, P and T; *L1+/y*: 1.19 ± 0.14, n = 70 boutons, 1 mouse; *L1-/y*: 1.25 ± 0.13, n = 57 boutons, 1 mouse; t-test, p= 0.66).

Scale bars: 100 μm in E; 2 μm in F, G, J and K. All data reported as mean ± SEM.

Supplemental Table 1

transcript	motor neurons	DRG neurons	prop neurons
Cntn1	+++	+++	+
Cntn2/TAG-1	+	++	+
Cntn3/BIG-1	--	+	-
Cntn4/BIG-2	+	+	np
Cntn5/NB2	-	++	+
Cntn6/NB3	-	-	-
Kirrel/Neph1	--	+	-
Kirrel-2/Neph3	--	-	
Kirrel-3/Neph2	-	++	+
Nephrin/Nphs1	-	-	
Nephrin2	-	-	
L1	+++	+++	-
CHL1	+	+	-
Neurofascin	++	++	+
NrCAM	++	++	-
Neuroligin1	-	-	
Neuroligin2	++	++	
Neuroligin3	++	++	
Neurexin	+	+	
Sidekick1	+	+	
Sidekick2	+	+	
Nectin1/Pvrl1	+	++	
Nectin2/Pvrl2	--	-	
Nectin3/Pvrl3	+	+	
Nectin4/Pvrl4	-	-	
Nectin-like1/Igsf4b/Cadm3	+++	+++	
Nectin-like2/Igsf4a/Cadm1	+	++	
Nectin-like3/Igsf4d/Cadm2	+	+	
Nectin-like4/Igsf4c/Cadm4	++	++	
Nectin-like5/Pvr	-	-	
Opcml	+	+	
Lsamp	+	+	
Negr1	+	+	
Cepu1/Ntm/Hnt	++	++	
DsCAM	+	+	
DsCAM-like	-	-	
NCAM	+	++	
NCAM2	--	-	
BIT/Sirpa	+	+	
CD47	++	+++	
ADAM23	++	++	
BEM1	+	+	
GPR116	-	-	
GPR124	++	+++	
GPR125	+	+	

Supplemental Table 1, related to Figure 1: Expression profile of Ig superfamily genes in motor neurons and DRG

Ig superfamily member transcript expression in p5 to 6 DRG and spinal cord. Proprioceptive expression was assessed using double labeling with Pv transcript and/or protein. Genes not expressed by motor neurons, but enriched in DRG or proprioceptive (prop) neurons are highlighted in orange and pink respectively (-- to +++: increasing expression levels; np: not processed).

SUPPLEMENTAL EXPERIMENTAL PROCEDURES

Quantitative Modeling

To further understand GABApre-sensory synaptic organization in the *NB2* mutant, we sought to quantitatively model the changes in GABApre bouton density per sensory afferent terminal in *NB2* mutant mice. Changes in GABApre-sensory synaptic organization were represented as a state diagram, where each state corresponds to the number of GABApre boutons on a sensory afferent terminal.

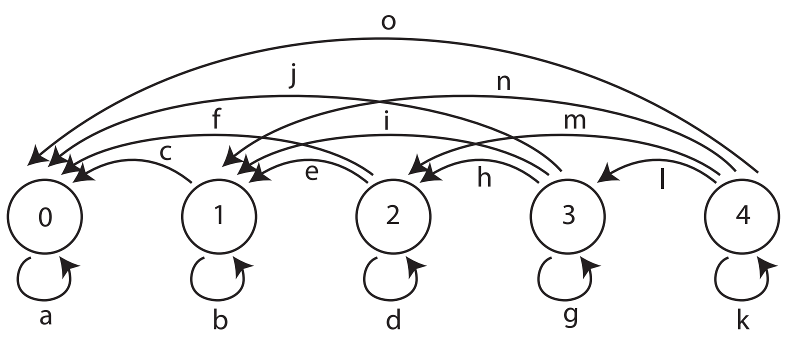

The arrows from each state represent the probability of transitioning from one state to another with loss of *NB2*. For example, if a wild type sensory afferent terminal is capable of accommodating 3 GABApre boutons (state 3), then there is a probability of *h* that it will accommodate 2 GABApre boutons (state 2) in *NB2* mutant mice.

From this state diagram, we can define the transition matrix *P* as

$$P = \begin{bmatrix} P(0,0) & P(0,1) & P(0,2) & \cdots & P(0,10) \\ 0 & P(1,1) & P(1,2) & \cdots & P(1,10) \\ 0 & 0 & P(2,2) & \cdots & P(2,10) \\ \vdots & \vdots & & \ddots & \vdots \\ 0 & 0 & 0 & & P(10,10) \end{bmatrix}$$

where $P(i, j)$ is the probability of transitioning from state j to state i with loss of NB2. Hypothesizing that it is unlikely for most GABApre boutons to be lost in the *NB2* mutant, we imposed the restriction that sensory afferent terminals may only be permitted to lose up to three GABApre boutons. This gives the transition matrix P

$$P = \begin{bmatrix} P(0,0) & P(0,1) & P(0,2) & P(0,3) & 0 & 0 & 0 & 0 & 0 & 0 & 0 \\ 0 & P(1,1) & P(1,2) & P(1,3) & P(1,4) & 0 & 0 & 0 & 0 & 0 & 0 \\ 0 & 0 & P(2,2) & P(2,3) & P(2,4) & P(2,5) & 0 & 0 & 0 & 0 & 0 \\ 0 & 0 & 0 & P(3,3) & P(3,4) & P(3,5) & P(3,6) & 0 & 0 & 0 & 0 \\ 0 & 0 & 0 & 0 & P(4,4) & P(4,5) & P(4,6) & P(4,7) & 0 & 0 & 0 \\ 0 & 0 & 0 & 0 & 0 & P(5,5) & P(5,6) & P(5,7) & P(5,8) & 0 & 0 \\ 0 & 0 & 0 & 0 & 0 & 0 & P(6,6) & P(6,7) & P(6,8) & P(6,9) & 0 \\ 0 & 0 & 0 & 0 & 0 & 0 & 0 & P(7,7) & P(7,8) & P(7,9) & P(7,10) \\ 0 & 0 & 0 & 0 & 0 & 0 & 0 & 0 & P(8,8) & P(8,9) & P(8,10) \\ 0 & 0 & 0 & 0 & 0 & 0 & 0 & 0 & 0 & P(9,9) & P(9,10) \\ 0 & 0 & 0 & 0 & 0 & 0 & 0 & 0 & 0 & 0 & P(10,10) \end{bmatrix}$$

To assess GABApre bouton maintenance in the *NB2* mutant, we parameterized the columns of P as

$$P = \begin{bmatrix} \beta_0 & 1-\beta_1 & \frac{1-\beta_2}{2} & \frac{1-\beta_3}{3} & 0 & 0 & 0 & 0 & 0 & 0 & 0 \\ 0 & \beta_1 & \frac{1-\beta_2}{2} & \frac{1-\beta_3}{3} & \frac{1-\beta_4}{3} & 0 & 0 & 0 & 0 & 0 & 0 \\ 0 & 0 & \beta_2 & \frac{1-\beta_3}{3} & \frac{1-\beta_4}{3} & \frac{1-\beta_5}{3} & 0 & 0 & 0 & 0 & 0 \\ 0 & 0 & 0 & \beta_3 & \frac{1-\beta_4}{3} & \frac{1-\beta_5}{3} & \frac{1-\beta_6}{3} & 0 & 0 & 0 & 0 \\ 0 & 0 & 0 & 0 & \beta_4 & \frac{1-\beta_5}{3} & \frac{1-\beta_6}{3} & \frac{1-\beta_7}{3} & 0 & 0 & 0 \\ 0 & 0 & 0 & 0 & 0 & \beta_5 & \frac{1-\beta_6}{3} & \frac{1-\beta_7}{3} & \frac{1-\beta_8}{3} & 0 & 0 \\ 0 & 0 & 0 & 0 & 0 & 0 & \beta_6 & \frac{1-\beta_7}{3} & \frac{1-\beta_8}{3} & \frac{1-\beta_9}{3} & 0 \\ 0 & 0 & 0 & 0 & 0 & 0 & 0 & \beta_7 & \frac{1-\beta_8}{3} & \frac{1-\beta_9}{3} & \frac{1-\beta_{10}}{3} \\ 0 & 0 & 0 & 0 & 0 & 0 & 0 & 0 & \beta_8 & \frac{1-\beta_9}{3} & \frac{1-\beta_{10}}{3} \\ 0 & 0 & 0 & 0 & 0 & 0 & 0 & 0 & 0 & \beta_9 & \frac{1-\beta_{10}}{3} \\ 0 & 0 & 0 & 0 & 0 & 0 & 0 & 0 & 0 & 0 & \beta_{10} \end{bmatrix}$$

where $\beta_i = P(i, i)$. Note that the parameters β_i represent the probability of having the same number of GABApre boutons on a sensory afferent terminal in the *NB2* mutant. We interpreted these parameters as measurements of GABApre-sensory synaptic stability. Because the entries in P are conditional probabilities, from the law of total probability, we can write

$$\begin{aligned} M_0 &= \beta_0 W_0 + (1-\beta_1)W_1 + \frac{(1-\beta_2)}{2}W_2 + \frac{(1-\beta_3)}{3}W_3 \\ M_1 &= \beta_1 W_1 + \frac{(1-\beta_2)}{2}W_2 + \frac{(1-\beta_3)}{3}W_3 + \frac{(1-\beta_4)}{3}W_4 \\ &\vdots \\ M_9 &= \beta_9 W_9 + \frac{(1-\beta_{10})}{3}W_{10} \\ M_{10} &= \beta_{10} W_{10} \end{aligned}$$

where M_i is the probability of observing i GABApre boutons on a sensory afferent

terminal in *NB2* mutants, and W_j is the probability of observing j GABApre boutons on a sensory afferent terminal in wild type mice. The values M_i and W_j are known from the experimental GABApre density distributions. Imposing the constraint that β_i must be between zero and one for all i, we can solve for the β_i parameters using a least squares approach (Matlab function *lsqlin*). We also tested the case in which sensory afferent terminals are permitted to lose all GABApre boutons and found that this returned comparable values for the β_i parameters.
